# Pharmacokinetics of Antibiotics in Crocodiles: A Review

**DOI:** 10.3390/ani15101363

**Published:** 2025-05-08

**Authors:** Seavchou Laut, Saranya Poapolathep, Pandaree Sitthiangkool, Narumol Klangkaew, Napasorn Phaochoosak, Mario Giorgi, Elena Badillo, Elisa Escudero, Pedro Marín, Amnart Poapolathep

**Affiliations:** 1 Department of Pharmacology, Faculty of Veterinary Medicine, Kasetsart University, Bangkok 10900, Thailand; seavchou.l@ku.th (S.L.); fvetsys@ku.ac.th (S.P.); pandaree.si@ku.th (P.S.); fvetnak@ku.ac.th (N.K.); fvetanp@ku.ac.th (N.P.); 2 Department of Veterinary Science, University of Pisa, 56112 Pisa, Italy; mario.giorgi@unipi.it; 3 Department of Pharmacology, Faculty of Veterinary Medicine, University of Murcia, 30100 Murcia, Spain; ebp2@um.es (E.B.); escudero@um.es (E.E.)

**Keywords:** antimicrobials, pharmacokinetics, dosage regimens, crocodiles

## Abstract

This review aims to provide an overview of the pharmacokinetics of antibiotics in crocodilian species, focusing on species-specific variations in drug absorption, distribution, metabolism, and elimination (ADME), as well as the influence of environmental factors. A review of the available literature across crocodilian species reveals notable pharmacokinetic variability. Environmental influences, such as temperature and metabolic rate, are shown to impact these pharmacokinetic parameters significantly. Despite the frequent use of antibiotics in clinical and conservation settings, the lack of standardized dosing regimens presents risks of under- or over-dosing. This variability is compounded by limited research on species-specific drug metabolism and elimination processes. The review highlights the need for further pharmacokinetic studies to develop evidence-based dosing protocols, optimize therapeutic outcomes, and address concerns related to antimicrobial resistance. Future research should focus on filling the gaps in PK data to refine dosing strategies and ensure both efficacy and safety in crocodilian species.

## Simple Summary

Antibiotics are used to treat bacterial infections in crocodiles; however, determining appropriate dosing regimens remains challenging due to interspecies variations in pharmacokinetics and the limited availability of pharmacological data. This review critically examined the commonly used antibiotics, their therapeutic efficacy, and the factors influencing optimal dosage. It identified the need for further pharmacokinetic (PK) and pharmacodynamic (PD) research to refine dosing strategies, thereby improving treatment outcomes and minimizing the risk of adverse effects.

## 1. Introduction

Antibiotics constitute a group of antimicrobial therapies that are the most commonly medically managed in reptiles [1]. The Reptilia class comprises a diverse range of species, which is more challenging than in mammals due to the existence of a variable pharmacological response to each chemotherapeutic agent. This is based on the broad range of behavioral, anatomic, and physiological characteristics exhibited by the various species. In contrast to mammals, reptiles are ectothermic, with physiological and biochemical processes being strongly influenced by their body temperature [2].

In this context, the family Crocodylidae, classified as semi-aquatic reptiles, has anatomical and physiological characteristics that distinguish it from mammals and birds, such as the ectothermy previously mentioned. This family has gained increasing importance in farming for production and consumption purposes. Crocodile farming has become an industry of great economic importance due to the high demand for skins, meat, and other by-products. Countries such as Thailand, Vietnam, China, India, and Cambodia have developed specialized farms for international trade, with the global market primarily focused on luxury fashion and gastronomy [3].

Crocodile farming in Asia generates thousands of direct and indirect jobs, ranging from farm management to the processing and commercialization of derived products. In many rural areas, this activity represents a key source of income, particularly for communities with limited access to other economic opportunities.

From a traditional perspective, most experience in animal production has been concentrated on mammals and birds, often leading to the extrapolation of management, disease prevention, and treatment measures from these species to crocodiles, which may pose considerable challenges.

Farm management practices are constantly evolving, driven by scientific research and the growing demand from manufacturers and consumers to ensure that supply chains comply with robust production criteria and scientifically backed animal welfare standards.

It is crucial to consider the unique physiological characteristics of reptiles to improve therapeutic outcomes in their pharmacological treatment for disease management or prevention. The effectiveness of therapeutic treatments in reptiles is determined by a thorough understanding of the physiological particularities of ectothermic organisms and the temperature conditions required for the metabolism and digestion of drugs and food [4].

Although some pharmacokinetic studies of antimicrobials in crocodiles have been carried out, there is still a need to establish well-founded hypotheses on their metabolism and immune response, adapted to each species and its specific conditions, to achieve therapeutic success while reducing the risk of bacterial resistance. Currently, studies on the pharmacokinetics of antibiotics in crocodiles are limited; typically, treatment regimens are extrapolated from other reptilian or mammalian studies using metabolic scaling calculations.

In this context, the aim of the current article was to review the existing literature on PK and PD studies of antimicrobials in crocodiles, to compile the available information and, where possible, recommend evidence-based doses and administration regimens.

## 2. Antibiotic Use in Reptiles

### 2.1. Aminoglycosides

Aminoglycosides are potent and broad-spectrum antibiotics that act through the inhibition of protein synthesis. They include kanamycin, gentamicin, amikacin, tobramycin, neomycin, and streptomycin [5]. Despite not representing a novel class of antimicrobials, aminoglycosides have demonstrated consistent clinical efficacy in combating infection. These are bactericidal antibiotics with a broad spectrum of activity, effective against both Gram-positive and Gram-negative bacteria [6]. Their use in reptiles is recommended in combination with penicillins and cephalosporins due to their synergistic effect, which enhances efficacy. However, these antibiotics are ineffective against anaerobic bacteria; furthermore, their action is limited in environments with a high protein load, such as abscesses and exudates. Additionally, their penetration into the central nervous system (CNS) and ocular tissues is poor, restricting their usefulness in infections affecting these areas [7].

A critical consideration in aminoglycoside therapy is their nephrotoxic potential, particularly in dehydrated reptiles or those with compromised renal function. This nephrotoxicity is exacerbated with prolonged use or inappropriate dosing, partly due to relatively inefficient renal excretion in crocodiles and other reptiles, which can lead to drug accumulation in the kidneys [7]. In such cases, adjustments or the selection of a less nephrotoxic drug are recommended to minimize risks and ensure safe treatment.

The pharmacokinetics of gentamicin sulfate and amikacin sulfate at two dosage levels have been reported in juvenile American alligators [8]. The alligators received gentamicin sulfate at 1.25 and 1.75 mg/kg body weight (b.w.) and amikacin sulfate at 1.75 and 2.25 mg/kg b.w. via a single intramuscular (i.m.) injection in the right forelimb. Based on the results, both gentamicin and amikacin were rapidly absorbed following i.m. administration at both dosage levels and showed biphasic serum concentration–time profiles. No substantial differences were observed between the serum concentrations and volume of distribution of gentamicin and amikacin administered at 1.75 mg/kg b.w. However, gentamicin showed significantly reduced clearance at the higher dose, while amikacin pharmacokinetics remained consistent across doses. The minimum inhibitory concentration (MIC) value for *A. hydrophila* in alligators is <6 μg/mL for gentamicin and 12.0 μg/mL for amikacin [8]. Based on these results, higher doses of the antimicrobial agents (gentamicin at 1.75 mg/kg and amikacin at 2.25 mg/kg) suggest potential clinical efficacy. However, formal PK/PD analysis and clinical outcomes were not assessed, so these conclusions remain tentative. An optimal reference dosage regimen for the American alligator has been established based on the pharmacokinetic data collected. Intramuscular administration of gentamicin at 1.75–2.25 mg/kg every 72–96 h and amikacin at 2.25 mg/kg every 72 h is recommended to ensure effective antimicrobial therapy [8]. No evidence of toxicity was observed in the course of that study. However, as the authors noted, toxicity studies of amikacin have not been reported in alligators or other reptiles, and only a few have been performed for gentamicin in snakes. Therefore, recommendations based on scientific studies are not yet available for crocodilians [8]. Table 1 provides a summary of the pharmacokinetic parameters for various aminoglycosides.

This study underscored the importance of adapting antibiotic protocols specifically for crocodilians. Gentamicin and amikacin showed extended half-lives in juvenile American alligators, indicating that longer dosing intervals (up to every 96 h) are both effective and safer. The serum concentrations achieved were adequate to treat *Aeromonas hydrophila*, a common bacterial pathogen in these animals. However, as highlighted by the authors, scientific data on amikacin toxicity in reptiles are lacking, and current dosage recommendations for crocodilians are based on limited evidence, and the safety of repeated dosing has not been fully established. Due to the potential for nephrotoxicity, it is essential to monitor hydration and kidney function, particularly in warmer environments. These results provide a foundation for safer, evidence-based aminoglycoside use in crocodilian medicine [8,9].

### 2.2. Beta-Lactams

Beta-lactams (β-lactams) are the most widely used class of antibiotics. The bactericidal mechanism of killing by β-lactams is recognized to be a major advantage for the treatment of serious infections [10]. The antibacterial effect of β-lactam antibiotics results from their inhibition of the final stage of peptidoglycan synthesis. They bind to acylate transpeptidases, also known as penicillin-binding proteins, which serve as the primary targets of β-lactams. This inhibition prevents proper cell wall formation, leading to bacterial death and lysis, due to the activation of autolytic enzymes [11].

#### 2.2.1. Penicillins

Penicillins are bactericidal antibiotics commonly used in veterinary medicine and effective against infections caused by Gram-positive cocci, Gram-positive rods, anaerobic bacteria, and Gram-negative cocci [12]. They are well distributed in extracellular spaces, particularly in inflamed tissues, making them highly effective for treating infections localized to such areas.

However, their ability to penetrate the CNS and the eye is limited, which restricts their use in infections affecting these structures. In terms of elimination, penicillins are primarily excreted by the kidneys in an unchanged form, resulting in high concentrations in the urine, which makes them particularly effective in treating urinary tract infections [13].

Additionally, penicillins exhibit a synergistic effect when combined with aminoglycosides, enhancing their bactericidal activity and broadening their spectrum of action against various Gram-negative bacterial pathogens. This synergy is based on the ability of penicillins to weaken the bacterial cell wall, thereby facilitating the penetration and activity of aminoglycosides within the microorganism [13].

One study focused on investigating the pharmacokinetics of amoxicillin trihydrate in Siamese freshwater crocodiles (*Crocodylus siamensis*) following intramuscular administration at doses of 5 and 10 mg/kg [14]. The average percentage of amoxicillin binding to plasma proteins was approximately 21.24%. Based on the pharmacokinetic findings, it was suggested that administering amoxicillin intramuscularly at a dose of 5 mg/kg body weight every 4 days could be a suitable treatment for susceptible bacterial infections in freshwater crocodiles, considering the susceptibility breakpoint and the surrogate PK/PD index (T > MIC, for a MIC less than or equal to 0.25 μg/mL).

Amoxicillin trihydrate demonstrated slow elimination and prolonged activity in freshwater crocodiles, with effective plasma levels maintained up to 72 h at a 10 mg/kg dose. A 5 mg/kg dose could be administered every 4 days, providing a practical and effective dosing regimen. These results help reduce stress from frequent injections and inform safer, targeted treatment strategies [14]. However, this theoretical dosing regimen needs to be confirmed by determining the MIC values of bacterial pathogens specifically affecting freshwater crocodiles to ensure the proposed dose’s clinical effectiveness [14]. Given the observed differences in amoxicillin pharmacokinetics between freshwater crocodiles and other animal species, dosing strategies in crocodilians should be guided by species-specific kinetic data that reflect underlying physiological, anatomical, and metabolic characteristics [15]. The study highlighted the critical role of pharmacokinetic research in guiding rational antimicrobial use in exotic species, especially crocodilian species. The pharmacokinetic data of amoxicillin trihydrate in freshwater crocodiles are summarized in Table 2.

#### 2.2.2. Cephalosporins

Cephalosporins are a large group of β-lactam antibiotics that are closely related to penicillins. They share the same mechanism of action as penicillin and exert their bactericidal effect by disrupting the bacterial cell wall in both Gram-negative and Gram-positive bacteria [16]. Cephalosporins are divided into five generations based on their spectrum of coverage against bacteria and temporal discovery [17].

Cephalosporins are antibiotics that are effectively distributed in extracellular spaces, allowing them to reach adequate concentrations in inflamed tissues, where infections are often localized. However, their ability to penetrate the eye and CNS is limited, with the exception of certain derivatives, such as cefotaxime and ceftazidime, which show a greater ability to cross these barriers. Additionally, cephalosporins can have a synergistic effect when combined with aminoglycosides, potentially enhancing their efficacy against the Gram-negative range of bacterial infections [1].

A study was conducted to evaluate the pharmacokinetic profile of ceftriaxone in freshwater crocodiles (*Crocodylus siamensis*) after i.m. administration at two dose levels of 12.5 and 25 mg/kg b.w. [18]. The t_1/2λz_, V_d_, and Cl values of ceftriaxone at the dosage of 12.5 mg/kg b.w. were 21.14 ± 3.72 h, 2800 ± 350 mL/kg, and 93 ± 8 mL/h/kg, respectively, which showed kinetic variation from the administration at the dosage of 25 mg/kg b.w. (19.93 ± 2.58 h, 3080 ± 430 mL/kg, and 108 ± 9 mL/h/kg, respectively). Notably, the Vd values of ceftriaxone observed in that study were higher than the typical Vd values of β-lactam antibiotics in mammals, which are usually less than 1000 mL/kg [19]. This difference suggests that ceftriaxone can achieve a wider distribution, allowing it to penetrate peripheral tissues and potentially reach intracellular compartments more effectively than other β-lactams. Its higher Vd value could be attributed to a relatively greater affinity for tissue binding sites and enhanced penetration into extracellular fluids [19]. This might also be due to reptiles having a comparatively low rate of body fluid transfer between the vascular and extravascular compartments and an estimated limited removal capacity of the ceftriaxone from the body system [18]. Adverse effects occurred, such as nervous disorders, excitement, tremors, and death, after intravenous administration of 12.5 mg/kg of ceftriaxone [18]. In comparison, after intramuscular injection, no adverse effects at the injection site and no behavioral or health alterations were found in animals with both dosages of ceftriaxone. The average percentage of protein binding of ceftriaxone in freshwater crocodiles was 53.78%. This value was different from that of mammalian species, which were in the range of 29–45% [20,21,22].

The study revealed that ceftriaxone, when administered i.m, maintained effective concentrations for 2–4 days in freshwater crocodiles, making it a viable option for treating bacterial infections. A 12.5 mg/kg dose may be sufficient for infections caused by organisms with a MIC ≤ 0.2 µg/mL. However, i.v. use led to fatal toxicity and should be avoided entirely in this species. In addition, the potential for allergic reactions or issues related to excipients must be considered when using this drug. Incorporating this drug into practice with proper dosing offers a valuable tool in managing bacterial disease while preserving animal welfare in a farm or clinical setting.

Another study evaluated the pharmacokinetics of ceftiofur intramuscularly at a dose of 30 mg/kg in six healthy juvenile American alligators (*Alligator mississippiensis*) [23]. The mean C_max_ was 23.2 µg/mL (range 16.0–27.9 µg/mL), the mean T_max_ was 72 h (range 72–120 h), and the mean terminal half-life was 143 h (range 90.8–220 h). Taking into account the differences between the two species, the T_max_ and t_½λz_ values for ceftiofur in alligators were much longer than those obtained with ceftriaxone in freshwater crocodiles [18], indicating a delayed absorption but also a slow distribution and elimination process from the body. Until the last sampling at 366 h, ceftiofur concentrations were above 2.0 µg/mL. Due to such prolonged plasma concentrations, a dosing interval could not be established in that study. The pharmacokinetic profiles of different cephalosporins in crocodilians are presented in Table 3.

### 2.3. Fluoroquinolones

Fluoroquinolones are bactericidal antibiotics with a broad spectrum of activity, effective against most Gram-negative pathogens, some Gram-positive species, the majority of *Mycoplasma* species, and potentially *Chlamydia*. However, their effectiveness is limited against anaerobic bacteria [24,25]. These antibiotics achieve high concentrations in various tissues, particularly in the liver and urinary tract, where tissue levels can exceed those found in plasma, making them particularly useful for treating localized infections in these areas.

Despite their therapeutic potential, fluoroquinolones should be used with caution in juvenile reptiles, as they have been associated with the risk of causing permanent joint defects [26]. Additionally, intramuscular injections of fluoroquinolones can result in substantial pain and necrosis at the injection site, emphasizing the need for careful injection techniques and monitoring [27].

Pharmacokinetic studies of enrofloxacin in estuarine crocodiles (*Crocodylus porosus*) were performed following a single dose of 5 mg/kg b.w. by i.v., i.m., and p.o. administration [28]. Following i.v. administration, enrofloxacin achieved values for an extrapolated C_0_ of 179.57 ± 55.43 µg/mL at time zero, for Vd_ss_ of 343.38 ± 64.70 mL/kg, for the Cl rate of 24.49 ± 1.26 mL/h/kg, and for the elimination half-life of 40.48 ± 5.70 h. Following i.m. and p.o. administration, the t_½λz_ were 19.02 ± 2.80 h and 64.98 ± 21.14 h, respectively. The systemic bioavailability after i.m. and p.o. administrations were 87.97 ± 12.36% and 142.50 ± 20.94%, respectively. For the PK/PD surrogate markers, the study reported an AUC-to-MIC ratio higher than 125:1 and a C_max_-to-MIC ratio higher than 8:1 for microorganisms with MIC values ≤0.5 µg/mL, then suitable effective concentrations are achievable lasting for 72 h based on i.v. and i.m. doses. Therefore, a dose of enrofloxacin (5 mg/kg b.w.) at least every 3 days after i.v. and i.m. administration should be effective against susceptible microorganisms. In estuarine crocodiles, enrofloxacin pharmacokinetic parameters were consistently lower than those observed in freshwater crocodiles despite identical dosing and administration route. The reported values of Vd_ss_ and Cl following i.v. administration in freshwater crocodiles were 3010 ± 1060 mL/kg and 40 ± 18 mL/g/kg, respectively. Similar values have been reported in American alligators, indicating comparable pharmacokinetic behavior in terms of drug distribution and clearance [29]. However, the mean t_½λz_ value obtained exceeded the previously reported values of 21 h i.v. in American alligators [30] and 40.5 h i.v. in estuarine crocodiles [28]. The observed differences could be influenced by species-specific factors, environmental temperature at the time of the experiment, the duration of blood sampling, or the sensitivity of the analytical method [29].

Enrofloxacin and ciprofloxacin were detected in the plasma of the freshwater crocodile, *C. siamensis*, after single i.v. or i.m. administrations at a dosage of 5 mg/kg b.w. Based on the experimental pharmacokinetic data, the single administration of ENR at a dosage of 5 mg/kg b.w. (i.m.) appeared to be appropriate for the treatment of susceptible bacteria (MIC 1.0 g/mL). For *C. siamensis*, maintaining steady-state levels with once-daily dosing would require 10–12 days, posing a challenge for clinical use [29].

Pharmacokinetic studies in freshwater crocodiles, estuarine crocodiles, and American alligators consistently showed that a 5 mg/kg dose of enrofloxacin can achieve therapeutic plasma concentrations suitable for treating infections caused by susceptible bacteria. Effective plasma concentrations are maintained for 36 to 72 h, depending on the species. These results suggested that less frequent dosing (every 48–72 h) could be both effective and less stressful for animals [28,29]. Oral administration, however, presents limitations due to delayed or erratic absorption, reinforcing intramuscular injection as the preferred route for acute treatment [30].

A pharmacokinetic study of marbofloxacin in freshwater crocodiles (*Crocodylus siamensis*) investigated the drug’s behavior following both i.v. and i.m. administration of a single dose of 2.0 mg/kg b.w. [31]. The t_½λz_ value was almost 60 h, showing that the rate of elimination of marbofloxacin in this species was slow. Furthermore, it was longer than in other studies using other species of reptiles (2.86–19.02 h) [32,33]. The Vd in freshwater crocodiles was 1494 mL/kg, and the Cl rate was 22.6 mL/h/kg. Differences between species or environmental temperatures might have different effects. Absolute i.m. bioavailability was very good at 105.39%. In the present study [32], the C_max_-to-MIC ratio was >10 for bacteria with a MIC value ≤ 0.25 µg/mL, whereas, in contrast, the AUC-to-MIC ratio was >125 with bacteria with a MIC ≤ 0.56 µg/mL. Fluoroquinolones, such as enrofloxacin, have demonstrated efficacy against a range of bacterial pathogens in crocodilians, including *Aeromonas* spp., *Pseudomonas* spp., *Salmonella* spp., and *Escherichia coli*, with the MIC values for these pathogens generally in the range ≤0.25–0.56 µg/mL [34,35]. In contrast, the MIC values of enrofloxacin against the most susceptible Gram-negative, Gram-positive, and Mycoplasma isolates in domestic animals are typically <0.1 µg/mL, with moderately susceptible strains showing MIC values between 0.125 and 0.5 µg/mL [36]. Therefore, a marbofloxacin dose of 2 mg/kg b.w. following i.v. and i.m. administration may be effective in treating bacterial infectious diseases in freshwater crocodiles [31]. When compared within estuarine crocodiles (*Crocodylus porosus*), the study of marbofloxacin pharmacokinetics at two different dosages of 2 and 4 mg/kg b.w. following i.m. administration [37], the results showed that the elimination half-life values were long (33.99 and 39.28 h) and were not significantly different. This half-life was notably longer than values reported in some other reptilian species [32,38,39,40,41,42]. These differences may be attributed to variation in metabolic pathways between species, as well as differences in body size, organ function, and environmental conditions, which can affect drug absorption, distribution, and elimination [43]. For the PK/PD surrogates, the AUC_0–24_-to-MIC ratio was >125 for MIC values lower than 0.125 and 0.35 µg/mL for doses of 2 and 4 mg/kg, respectively. Furthermore, it is likely to be effective against several bacterial pathogens with a MIC < 0.35 µg/mL for the dosage of 4 mg/kg b.w. after i.m administration in estuarine crocodiles [37]. These findings highlighted the potential of marbofloxacin as an effective treatment for bacterial infections in crocodiles. The prolonged half-life and high bioavailability support less frequent dosing, which is advantageous in managing large or potentially dangerous animals. Based on PK/PD targets, marbofloxacin at 2–4 mg/kg may achieve therapeutic success against pathogens with MIC values below 0.35 µg/mL, which includes many clinically relevant isolates in crocodilians [31,37]. No adverse effects at the point of injection and no behavioral or health alterations were observed in the experimental animals during or after the study.

In freshwater crocodiles (*Crocodylus siamensis*), a pharmacokinetic study of danofloxacin was administered following a single i.m. injection at two different dosages of 6 and 12 mg/kg b.w. [44]. The results showed that the AUC and C_max_ values increased with the dosage. Following these two dosages, the t_½λz_ was long at 48.18 h and 67.29 h, respectively, with low Cl values. PK/PD surrogates, in that study were recorded after a single danofloxacin dose by i.m. of 6 mg/kg b.w., with the AUC_0–24_-to-MIC ratio being >125 with bacteria; thus, a MIC < 0.04 µg/mL appears to be suitable for the treatment of bacterial diseases [44]. The slower elimination of danofloxacin observed in freshwater crocodiles, relative to other reptilian species [45,46,47], could be attributed to interspecies physiological variation, environmental conditions, timing of blood collection, and differences in analytical techniques [15].

The authors noted that the small sample size and variability in danofloxacin plasma concentrations, potentially influenced by the parallel experimental design using different animal groups, may have contributed to this difference, and further studies are needed to confirm the findings [44]. The pharmacokinetic profile of danofloxacin at 6 mg/kg i.m. suggested this dose may be effective for treating infections caused by bacteria with MICs under 0.04 μg/mL in freshwater crocodiles. This provided a useful starting point for empirical therapy, though further research is needed to define an optimal, evidence-based dosing regimen for repeated administration [44].

### 2.4. Macrolides

Macrolides are a class of antimicrobial drugs, including azithromycin, clarithromycin, and erythromycin, that are used to manage and treat most bacterial infections. Commonly, they are used against bacterial infectious diseases such as pneumonia, pharyngitis, and sinusitis [48,49]. The mechanism of action of macrolides is by inhibiting protein synthesis in bacteria, binding to the 50S ribosomal subunit, and preventing the translation of mRNA, specifically the growing peptide chain by preventing the peptidyl transferase from adding the subsequent amino acid attached to the tRNA [48]. Ketolides, with an additional binding site, have a stronger ribosomal interaction. Some macrolides may also cause premature peptidyl tRNA dissociation, while erythromycin specifically interferes with 50S subunit assembly [50].

In freshwater crocodiles (*Crocodylus siamensis*) following a single dose of 2 mg/kg b.w. of clarithromycin after i.v. and i.m. administration [51], there were no significant differences among groups in the elimination half-lives. The values were 20 h for both routes, showing that the long elimination half-life was consistent with the low elimination rate constant and the slow clearance rate. That study suggested a dose of 2.5 mg/kg might be effective if administered once every 2 weeks against susceptible bacteria with a MIC of 0.5 µg/mL, where a dose of 2.5 mg/kg might be effective for at least 10 days [51]. The pharmacokinetic data for different fluoroquinolones in crocodilians are summarized in Table 4.

In the same species (*Crocodylus siamensis*), a pharmacokinetic study of azithromycin was administered at three different dosages of 2.5, 5, and 10 mg/kg b.w. following a single i.m. administration [52]. The study identified no significant differences among groups. The t_½λz_ values were long at 33.70, 38.11, and 34.80 h, respectively, for the three dosages. The AUC and C_max_ values increased with the dosages. For PK/PD surrogates, the T > MIC is an important predictor of efficacy for time-dependent drugs where plasma concentrations should be above the MIC for at least 40–50% of dosing intervals [53]. The MIC_90_ of azithromycin for *Aeromonas hydrophila*, the most common pathogen in respiratory diseases of reptiles is 4 µg/mL [54]. In that study, at the azithromycin dosages of 2.5, 5, and 10 mg/kg b.w. the concentrations above the MIC values were determined to last for 1, 6, and 24 h after i.m. administration, respectively. Therefore, a dose of 10 mg/kg b.w. might be effective against bacterial pathogens in freshwater crocodiles after i.m. administration every 48 h [52]. Similar pharmacokinetic studies on azithromycin have been conducted in reptiles, including ball pythons, where t_½λz_ values ranged from 17 h (i.v.) to 51 h (p.o.) at a 10 mg/kg b.w. dose [55]. These results are consistent with the prolonged half-life of azithromycin in freshwater crocodiles, but differences in pharmacokinetic profiles between species may arise due to varying metabolic processes, environmental conditions, or drug absorption rates [52].

The disposition kinetics of tildipirosin were examined in estuarine and freshwater crocodilians following i.v. (2 mg/kg) and i.m. (2 and 4 mg/kg) administrations [56]. The Vd post-i.v. dosing was notably lower in both crocodilian species compared to mammals [57,58,59,60], indicating limited tissue distribution. Intramuscular administration at 2 mg/kg showed complete bioavailability, although this decreased by 20–25% at a 4 mg/kg dose. Notably, the pharmacokinetics of tildipirosin varied substantially between the two crocodilian species. Based on a MIC of 0.5 μg/mL, the AUC_0–24_-to-MIC ratio suggested that tildipirosin would surpass the 65 h threshold for both crocodilian species at both dose levels, showing a bacteriostatic effect. Importantly, no adverse reactions with tildipirosin were reported post-treatment in either species. Given these findings and the favorable pharmacokinetic profile observed, tildipirosin represents a promising option for managing bacterial infections in estuarine and freshwater crocodilians [56].

The data indicated that clarithromycin, azithromycin, and tildipirosin could be integrated into treatment protocols based on their pharmacokinetic characteristics. Clarithromycin’s extended t_½λz_ makes it suitable for biweekly dosing at 2.5 mg/kg, ensuring that plasma concentrations remain effective against susceptible pathogens for a longer period [51]. Azithromycin, with an impressive t_½λz_ of 33–38 h, offers flexibility in dosing, with the 10 mg/kg dose providing sufficient coverage for up to 24 h, making it an excellent choice for treating *Aeromonas hydrophila* infections [52,53,54]. For tildipirosin, its favorable pharmacokinetic profile, combined with effective tissue penetration, makes it a promising antibiotic in treating a variety of bacterial infections, potentially reducing the frequency of dosing required while maintaining efficacy [56,61]. The pharmacokinetic parameters of different macrolides in crocodilians are summarized in Table 5.

### 2.5. Tetracyclines

The tetracyclines are bacteriostatic, broad-spectrum agents, which exhibit activity against a wide range of Gram-positive and Gram-negative bacteria, covering aerobic and anaerobic pathogens and intracellular bacteria such as *Mycoplasma* spp. and *Chlamydophila* spp. They exhibit antibacterial activity by targeting the 30S ribosomal subunit. This prevents aminoacyl-tRNA from attaching to the mRNA-ribosome complex, effectively blocking bacterial protein synthesis. They also target 70S ribosomes in mitochondria, interfering with mitochondrial protein production [62,63]. Due to their efficient absorption, low toxicity, and cost-effectiveness, tetracyclines are extensively used in treating bacterial infections in human and veterinary medicine. In some mammalian species, their enterohepatic recirculation extends their half-life, and elimination occurs mainly through glomerular filtration and biliary pathways [64]. The MIC values of oxytetracycline for most bacterial pathogens that can cause substantial diseases in animal species are about 0.25–2.0 μg/mL [65,66].

The pharmacokinetic parameters for the tetracycline group, as summarized in Table 6, including in freshwater crocodiles, a long-acting formulation of oxytetracycline was evaluated after i.m. administration at three different dosages of 5, 10, and 20 mg/kg b.w. [67]. The calculated kinetic parameters were t_1/2λz_ values of 33.59 ± 2.52 h, 38.42 ± 5.47 h, and 38.04 ± 1.98 h, respectively, while the C_max_ values were 2.15 ± 0.51 μg/mL, 7.68 ± 1.08 μg/mL, and 17.08 ± 2.09 μg/mL, respectively. The value for t_1/2λz_ was significantly shorter in the 5 mg/kg b.w. group than in the 10 and 20 mg/kg b.w. groups. The plasma protein-binding percentage for the average oxytetracycline was 32.69% ± 9.16%. The MRT values were shorter than those reported in American alligators [68].

The pharmacokinetics in American alligators were evaluated using single i.v. and i.m. injections of a long-acting oxytetracycline [69]. The alligators received long-acting oxytetracycline as a single 10 mg/kg i.v. bolus in the paravertebral vein. During i.v. administration of oxytetracycline, the mean C_0_ at the initial time was 60.63 ± 28.26 μg/mL, and mean drug levels were 2.82 ± 0.71 μg/mL at the end of the 192 h sampling period. The mean V_P_ was 200 ± 90 mL/kg with a mean elimination half-life (t_1/2λz_) of 15.15 h and a mean Cl of 7 ± 2 mL/h/kg. The mean peak plasma concentration was 6.85 ± 1.96 μg/mL at 1 h after i.m. administration, with a mean drug level of 4.05 ± 1.97 μg/mL at the end of the 192 h sampling period. The prolonged half-life of oxytetracycline following i.m. administration compared to i.v. administration suggests a depot effect, which gradually releases the drug into circulation. This extended release may have been due to the presence of pyrrolidine salt, which contributes to sustained drug release but may also cause localized irritation [68,69,70]. While i.v. administration can potentially lead to neuromuscular blockade and tetany [71] (including in the tetracycline group), no adverse effects were noted in that study. The C_max_ value after i.m. administration was considerably lower than the C_0_ value observed following i.v. administration, with the plasma concentration at 192 h being greater in the i.m. group, indicating that i.m. administration extended the therapeutic effect. These studies demonstrated that the pharmacokinetics of oxytetracycline in both freshwater crocodiles and American alligators are influenced by dosage. In freshwater crocodiles, higher dosages of 10 and 20 mg/kg b.w. resulted in significantly longer half-lives and increased plasma concentrations compared to the 5 mg/kg dose. This indicates that i.m. administration at these higher doses could provide prolonged therapeutic effects. The extended release of oxytetracycline observed in alligators following i.m. administration further highlights the potential of this approach in maintaining effective drug concentrations over time. Clinically, these findings suggest that a 10 or 20 mg/kg i.m. dose in freshwater crocodiles could offer sustained antibacterial effects, reducing the need for frequent dosing [70].

**Table 6:** Pharmacokinetic parameters of different tetracyclines in crocodilians.

Species	Drug	Dose (mg/kg)	AUC	AUC_last_	AUC_0–∞_ (μg × h/mL)	C_0_	C_max_ (μg/mL)	T_max_ (h)	Vd (mL/kg)	Cl (mL/h/kg)	t_½λz_ (h)	MRT	Information Source
Freshwater crocodiles	Long-acting formulation of oxytetracycline	5 (i.m.; n = 5)		199.27 ± 51.91	206.96 ± 54.56	-	2.15 ± 0.51	1.0	-	-	33.59 ± 2.51	76.36 ± 8.79	[68]
		10 (i.m.; n = 5)		1101.36 ± 164.54	1196.05 ± 159.93	-	7.68 ± 1.08	0.5	-	-	38.42 ± 25.47	93.55 ± 2.72	
		20 (i.m.; n = 5)		2405.15 ± 182.37	2611.76 ± 196.08	-	17.08 ± 2.09	0.5	-		38.04 ± 1.98	92.03 ± 3.01	
American alligators	Long-acting formulation of oxytetracycline	10 (i.v.; n = 6)	1468.85 ± 413.91	-	-	60.63 ± 28.26	-	-	790 ± 100	7 ± 2	15.15 ± 17.01	119.01 ± 59.75	[70]
American alligators	Tetracycline	50 (p.o.; n = 12)	41 ± 1.25 (Fasted) 15 ± 3 (Nonfasted)	-	-	-	0.52 ± 0.14 (Fasted) 0.46 ± 0.22 (Nonfasted)		10.7 ± 4.6 (Fasted) 9 ± 1.9 (Nonfasted)	-	-	-	[67]

Another study described the pharmacokinetic parameters of a single dose of tetracycline at 50 mg/kg after oral administration, via gastric gavage, in the American alligator [67]. That study on fasted and fed alligators reported C_max_ values of 0.52 ± 0.14 μg/mL and 0.46 ± 0.22 μg/mL, respectively, and T_max_ values of 10.7 ± 4.6 h and 9 ± 1.9 h, respectively. The mean absorption of the fasted alligators was 1.6 ± 1.2 h, and for the fed alligators, the mean absorption was 2.1 ± 1.2 h. The mean terminal elimination half-life of the fasted alligators (45 ± 26 h) was greater than in the fed alligators (21 ± 15 h) but not significantly so (P = 0.07). In a clinical context, the findings from this study suggested that the pharmacokinetics of tetracycline in American alligators are influenced by their nutritional state. Specifically, the longer t_½λz_ observed in fasted alligators indicated that the drug may stay in the system longer under fasting conditions, potentially providing extended antibacterial activity. However, since the difference between the fasted and fed groups was not statistically significant, this effect may not be clinically relevant in all situations [67].

Research in reptiles and other species has suggested that fasting can alter drug absorption and elimination, often delaying absorption and extending the duration of elimination. This effect is likely due to modifications in gastrointestinal motility and enzyme function, particularly cytochrome P450 (CYP) enzymes. These enzymes play a crucial role in drug metabolism; fasting can either increase or decrease the activity of certain CYP enzymes and phase II enzymes, affecting drug clearance and therapeutic effectiveness [72]. Although the findings in that study were not statistically significant, the data suggested that fasting may influence both absorption and elimination. Further investigation with larger sample sizes and more controlled conditions could provide deeper insights into the mechanisms behind these differences. The pharmacokinetic/pharmacodynamic (PK/PD) parameters and MIC values of all studied drugs are summarized in Table 7.

## 3. Summary

The number of published pharmacokinetic studies of antibiotic usage in crocodiles is increasing. Therefore, more information and data are becoming available, including dosing regimens, route of administration, treatment duration, and therapeutic effectiveness for patients. However, it is important to acknowledge that several of the studies reviewed involved limited sample sizes, which may affect the robustness and generalizability of the results. Future studies with larger and more diverse samples are needed to enhance the accuracy of pharmacokinetic predictions in crocodilians. Antibiotic therapy and selected appropriate agents should be considered to confirm therapeutic effectiveness, especially in the light of increasing antibacterial resistance. Pharmacokinetic differences among crocodilian species emphasize the complexity of how drugs are processed in these animals. Variations in metabolic rates, enzyme activity, body composition, and environmental factors contribute to the diverse PK profiles when developing veterinary treatments for crocodilians. This approach is essential for refining drug dosing guidelines and achieving effective therapeutic outcomes.

## Figures and Tables

**Table 1:** Pharmacokinetic parameters of different aminoglycosides in crocodilians.

Species	Drug	Dose (mg/kg)	C_max_ (μg/mL)	T_max_ (h)	Vd (mL/kg)	Cl (mL/h/kg)	t_1/2λz_ (h)	Information Source
American alligators	Gentamicin sulfate	1.25 (i.m.; *n* = 6)	-	-	387 ± 114	7.05 ± 1.49	37.8 ± 4.0	[8]
		1.75 (i.m.; *n* = 6)	9.5	0.5	287 ± 57	2.67 ± 0.63	75.4 ± 10.7	
	Amikacin sulfate	1.75 (i.m.; *n* = 6)	-	-	252 ± 66	3.64 ± 0.52	49.4 ± 18.2	
		2.25 (i.m.; *n* = 6)	14.5	1	224 ± 36	3.09 ± 0.79	52.8 ± 15.8	

**Table 2:** Pharmacokinetic parameters of amoxicillin trihydrate in freshwater crocodiles.

Drug	Dose (mg/kg)	AUC_last_ (μg × h/mL)	AUC_0–∞_ (μg × h/mL)	C_max_ (μg/mL)	T_max_ (h)	Vd (mL/kg)	Cl (mL/h/kg)	t_1/2λz_ (h)	MRT	K_el_ (1/h)	Information Source
Amoxicillin trihydrate	5 (i.m.; n = 5)	182.89 ± 47.86	184.07 ± 48.06	6.64 ± 1.76	8.4 ± 0.89	360 ± 80	29 ± 6	8.72 ± 0.61	21.54 ± 2.76	0.080 ± 0.005	[14]
	10 (i.m.; n = 5)	330.70 ± 70.03	331.22 ± 69.92	11.23 ± 2.40	10.0 ± 0.0	410 ± 140	31 ± 6	8.98 ± 1.13	23.49 ± 2.20	0.078 ± 0.008	

**Table 3:** Pharmacokinetic parameters of different cephalosporins in crocodilians.

Species	Drug	Dose (mg/kg)	AUC_last_	AUC_0–∞_ (μg × h/mL)	C_max_ (μg/mL)	T_max_ (h)	Vd (mL/kg)	Cl (mL/h/kg)	t_½λz_ (h)	MRT	K_el_ (1/h)	Information Source
Freshwater crocodiles	Ceftriaxone	12.5 (i.m.; n = 5)	135.73 ± 11.86	147.04 ± 17.09	24.61 ± 5.15	0.5	2800 ± 350	93 ± 8	21.14 ± 3.72	24.41 ± 4.46	0.034 ± 0.006	[18]
		25 (i.m.; n = 5)	234.31 ± 22.71	244.61 ± 24.07	26.39 ± 2.81	1.0	3080 ± 430	108 ± 9	19.93 ± 2.58	28.28 ± 2.46	0.035 ± 0.005	
American alligators	Ceftiofur crystalline- free acid	30 (i.m.; n = 6)	4.24	-	23.2	72	-	-	143	-	0.00480	[23]

**Table 4:** Pharmacokinetic parameters of different fluoroquinolones in crocodilians.

Species	Drug	Dose (mg/kg)	AUC_last_	AUC_0–∞_ (μg × h/mL)	C_max_ (μg/mL)	T_max_ (h)	Vd (mL/kg)	Cl (mL/h/kg)	t_½λz_ (h)	MRT	F (%)	K_el_ (1/h)	Information Source
Estuarine crocodiles	Enrofloxacin	5 (i.v.; n = 5)	1327.01 ± 117.35	1769.19 ± 12.51	-	-	160.92 ± 23.18	24.49 ± 1.26	40.48 ± 5.70	141.05 ± 17.25	-	-	[28]
		5 (i.m.; n = 5)	74.38 ± 12.95	133.23 ± 7.05	8.90 ± 2.28	0.65 ± 0.15	-	-	19.02 ± 2.80	10.19 ± 0.20	87.97 ± 12.36	-	
		5 (p.o.; n = 5)	1015.45 ± 63.35	1901.24 ± 299.18	21.04 ± 3.17	21.20 ± 7.03	-	-	64.98 ± 21.14	34.47 ± 1.81	142.50 ± 20.94	-	
Freshwater crocodiles	Enrofloxacin	5 (i.v.; n = 5)	107.75 ± 31.74	125.65 ± 39.37	-	-	3390 ± 1560	40 ± 18	43.24 ± 9.98	45.33 ± 4.46	-	0.01 ± 0.002	[29]
		5 (i.m.; n = 5)	86.38 ± 10.91	97.54 ± 5.41	2.33 ± 0.60	7.20 ± 1.79	-	-	44.72 ± 5.16	39.44 ± 5.19	82.65	0.02 ± 0.002	
American alligators	Enrofloxacin	5 (i.v.; n = 6)	119.10 ± 43.34 (AUC)	-	1.41 ± 0.37	-	1880 ± 960	47 ± 21	21.05 ± 11.84	77.04 ± 21.00	-	0.033 ± 0.025	[29]
		5 (p.o.; n = 5)	37.31 ± 18.86	201.02 ± 225.15 Extra% 56.62 ± 29.06	0.50 ± 0.27	55 ± 29	-	-	77.73 ± 4.84	139.58 ± 146.56	-	-	
Freshwater crocodiles	Marbofloxacin	2 (i.v.; n = 6)	-	97.29 ± 15.08	-	-	-	22.62 ± 4.10	57.51 ± 8.51	72.50 ± 10.92	-	0.01 ± 0.001	[31]
		2 (i.m.; n = 6)	-	102.91 ± 15.07	2.70 ± 0.52	0.54 ± 0.25	-	-	57.61 ± 11.79	75.06 ± 8.98	105.39	0.012 ± 0.002	
Estuarine crocodiles	Marbofloxacin	2 (i.m.; n = 5)	57.34	58.78	1.55	2	1668.8 (Vz/F)	34.02 (Cl/F)	33.99	49.0	-	0.02	[37]
		4 (i.m.; n = 5)	175.28	183.98	4.96	1	1231.9 (Vz/F)	21.74 (Cl/F)	39.28	56.29	-	0.02	
Freshwater crocodiles	Danofloxacin	6 (i.m.; n = 5)	213.17	235.72	7.20	0.30	1630 (Vd/F)	0.024 (Cl/F)	48.18	49.53	-	0.014	[44]
		12 (i.m.; n = 5)	493.96	611.91	12.30	0.35	1970 (Vd/F)	0.020 (Cl/F)	67.29	59.01	-	0.010	

**Table 5:** Pharmacokinetic parameters of different macrolides in crocodilians.

Species	Drug	Dose (mg/kg)	AUC_last_	AUC_0–∞_ (μg × h/mL)	C_max_ (μg/mL)	T_max_ (h)	Vd (mL/kg)	Cl (mL/h/kg)	t_½λz_ (h)	MRT	F (%)	K_el_ (1/h)	Information Source
Freshwater crocodile	Clarithromycin	2.5 (i.v.; n = 5)	149.15	163.48	-	-	-	0.017	20.65	27.13	-	0.033	[51]
		2.5 (i.m.; n = 5)	221.89	224.10	9.08	2.29	-	-	19.36	39.10	137.09	0.036	
Freshwater crocodiles	Azithromycin	2.5 (i.m.; n = 5)	82.99	86.24	8.21	0.5	-	-	33.70	40.92	-	0.021	[52]
		5 (i.m.; n = 5)	191.11	200.40	13.16	0.5	-	-	38.11	40.16	-	0.018	
		10 (i.m.; n = 5)	411.84	425.40	36.97	1	-	-	34.80	36.65	-	0.019	
Estuarine crocodile	Tildipirosin	2 (i.v.; n = 5)	331.32 ± 70.60	373.51 ± 69.71	-	-	360 ± 100	6 ± 1	44.22	63.91 ± 9.89	-	0.016 ± 0.002	[56]
		2 (i.m.; n = 5)	325.65 ± 30.83	392.67 ± 33.99	12.63 ± 1.65	1.60 ± 0.55	-	-	59.52	77.68 ± 6.58	108.00 ± 21.44	-	
		4 (i.m.; n = 5)	465.85 ± 89.82	554.96 ± 99.62	15.11 ± 2.11	1.40 ± 0.55	-	-	56.99	75.51 ± 9.55	76.71 ± 21.04	0.012 ± 0.002	
Freshwater crocodile	Tildipirosin	2 (i.v.; n = 5)	66.14 ± 11.11	70.71 ± 11.02	-	-	1480 ± 260	29 ± 4	35.33	45.61 ± 5.12	-	0.020 ± 0.002	
		2 (i.m.; n = 5)	68.42 ± 17.19	76.56 ± 20.43	6.44 ± 1.20	1.40 ± 0.55	-	-	44.50	56.62 ± 6.27	107.82 ± 22.52	0.016 ± 0.003	
		4 (i.m.; n = 5)	101.06 ± 9.38	111.07 ± 11.38	9.77 ± 1.43	1.40 ± 0.55	-	-	41.50	51.72 ± 8.36	80.20 ± 15.52	0.017 ± 0.003	

**Table 7:** Summary of pharmacokinetic/pharmacodynamic (PK/PD) parameters and MIC values.

Antibiotic	Species	PK/PD Index	MIC Target (µg/mL)	Achieved PK/PD	Information Source
Gentamicin	American alligator	C_max_/MIC ≥ 8	<6 (*A. hydrophila*)	C_max_ = 9.5 µg/mL (i.m. 1.75 mg/kg)	[8]
Amikacin	American alligator	C_max_/MIC ≥ 8	<12	C_max_ = 14.5 µg/mL (i.m. 2.25 mg/kg)	
Amoxicillin trihydrate	Freshwater crocodile	T > MIC ≥ 40–50%	≤0.25	Sustained for 96 h (5 mg/kg)	[14]
Ceftriaxone	Freshwater crocodile		≤1	Above MIC for 72–96 h	[18]
Ceftiofur	American alligator		≤2	Sustained > 366 h	[23]
Enrofloxacin	Estuarine crocodile	AUC/MIC ≥ 125; C_max_/MIC ≥ 8	≤0.5	Met targets via i.m. & i.v.	[28]
	American alligator			Effective via i.v.	[30]
	Freshwater crocodile		>1	Requires high doses	[29]
Marbofloxacin	Freshwater crocodile	AUC/MIC ≥ 125; C_max_/MIC ≥ 10	≤0.25–0.56	Achieved PK/PD thresholds	[31]
Danofloxacin	Freshwater crocodile	AUC/MIC ≥ 125	<0.04	Met targets at 6 mg/kg	[44]
Clarithromycin	Freshwater crocodile	T > MIC ≥ 40–50%	0.5	Sustained for 10 days at 2.5 mg/kg	[51]
Azithromycin	Freshwater crocodile		4 (*A. hydrophila*)	Met at 10 mg/kg for 24 h	[52]
Tildipirosin	Estuarine & Freshwater	AUC/MIC ≥ 65	0.5	Sustained > 65 h at both doses	[56]

## Data Availability

No new data were created or analyzed in this study. Data sharing is not applicable to this article. For further enquiries, contact Amnart Poapolathep; amnart.p@ku.th.
